# Temporal binding past the Libet clock: testing design factors for an auditory timer

**DOI:** 10.3758/s13428-020-01474-5

**Published:** 2020-10-15

**Authors:** Felicitas V. Muth, Robert Wirth, Wilfried Kunde

**Affiliations:** Department of Psychology, Julius-Maximilians-University of Würzburg, Röntgenring 11, 97070 Würzburg, Germany

**Keywords:** Temporal binding, Auditory timer, Experimental design, Measures, Intentional binding

## Abstract

Voluntary actions and causally linked sensory stimuli are perceived to be shifted towards each other in time. This so-called temporal binding is commonly assessed in paradigms using the Libet Clock. In such experiments, participants have to estimate the timing of actions performed or ensuing sensory stimuli (usually tones) by means of a rotating clock hand presented on a screen. The aforementioned task setup is however ill-suited for many conceivable setups, especially when they involve visual effects. To address this shortcoming, the line of research presented here establishes an alternative measure for temporal binding by using a sequence of timed sounds. This method uses an auditory timer, a sequence of letters presented during task execution, which serve as anchors for temporal judgments. In four experiments, we manipulated four design factors of this auditory timer, namely interval length, interval filling, sequence predictability, and sequence length, to determine the most effective and economic method for measuring temporal binding with an auditory timer.

## Introduction

Opening an app on an outdated smartphone typically comes with a slight and sometimes barely noticeable time interval between tapping the screen and opening of the app. However, the perceived time interval between tap and the presentation of the app’s content is shortened. More precisely, when the tap opens the app, the tap is judged to occur later, and the app is judged to flash earlier, as compared to situations where there is only a tap or only a flashing of an app. This so-called temporal binding phenomenon (also referred to as intentional binding) is widely employed in research on voluntary actions and their subsequent effects (Haggard, Clark, & Kalogeras, 2002; Moore & Obhi, 2012). It describes the finding that an action and a causally linked sensory event are perceptually shifted towards each other in time, as compared to either of the events happening in isolation. That is, if you tapped on the icon on your smartphone but the app did not open, you would have a more accurate temporal estimate of your action than if the app actually opened (though prediction of what will happen induces a small shift in perceived action time as well, Moore & Haggard, 2008). Likewise, if you watched your screen and an app opened without your involvement, you would have a more accurate estimate of the time the app opened than if you actively pressed an icon to open the app.

Due to the lack of explicit awareness of such perceptual shifts, temporal binding is an implicit measure for the sense of agency, i.e., the conception of the self as being responsible for our actions, and through these, changes in the environment (Haggard & Tsakiris, 2009; Moore, 2016; Tsakiris & Haggard, 2003). This sense of agency is informed by predictive and retrospective processes that reflect peoples’ feelings of agency and peoples’ judgments of agency, respectively (Sidarus, Vuorre, & Haggard, 2017; Synofzik, Vosgerau, & Voss, 2013). Temporal binding, which is sensitive to intentions but does not require explicit reflections regarding agency, is supposed to reflect predictive processes based on the agent’s internal sensorimotor models (Synofzik, Vosgerau, & Newen, 2008). On the contrary, Hughes, Desantis, and Waszak (2013) argue that temporal binding is rather driven by temporal expectancy and not intentional causation.

Beyond the fact that temporal binding is sensitive to intentions and is thus often referred to as intentional binding (e.g., Haggard & Tsakiris, 2009; Moore & Obhi, 2012), it has been shown that temporal binding is also informed by causality, which is why intentions are not a prerequisite for it to arise (Buehner, 2012; Suzuki, Lush, Seth, & Roseboom, 2019). It is a widely employed measure for time estimations in both healthy participants and clinical populations such as patients with schizophrenia or Parkinson’s disease (Buehner & Humphreys, 2009; Haggard, Martin, Taylor-Clarke, Jeannerod, & Franck, 2003; Kirsch, Kunde, & Herbort, 2019; Moore et al., 2010). Despite the common use of temporal binding as a measure, as of yet there are not many ways of studying it. Temporal binding is commonly assessed with two paradigms: interval estimation and the Libet Clock (Engbert, Wohlschläger, Thomas, & Haggard, 2007; Tanaka, Matsumoto, Hayashi, Takagi, & Kawabata, 2019). They are both based on the phenomenon that the perceived interval between voluntary self-generated actions and causally linked sensory events is shortened. However, the major difference is that in studies employing the interval estimation method, participants have to estimate the length of the interval between action and effect, while with the Libet clock, both the timing of the action and the timing of the effect have to be estimated independently.

In studies using the Libet Clock, participants have to estimate the timing of their actions and subsequent events by means of a so-called Libet Clock, which is presented on a screen. This clock is designed such that a full rotation of the clock hand takes about 2560 ms rather than 60 seconds. During the experiments, participants view the rotating clock hand while performing voluntary button presses and experiencing their effects (usually sounds). Subsequently, they report the position of the clock hand at specific occurrences. These occurrences are either the participants’ actions or the ensuing effects (for more detail see Fig. 2) (e.g., Libet, Gleason, Wright, & Pearl, 1983; Ruess, Thomaschke, & Kiesel, 2017b). Results show that voluntary actions are systematically perceived as having happened later, shifted towards the effect, when occurring in combination with a sensory event compared to when occurring in isolation (action binding). The same accounts for time estimations of effects following voluntary actions. Subsequent to self-generated actions, effects are judged to have occurred earlier, shifted towards the action, as compared to effects that happened in isolation (effect binding). Consequently, the interval estimation method can only make inferences about the overall binding, while the other method is capable of disentangling action binding and effect binding.

However, the use of the Libet Clock has several limitations as well. Pockett and Miller (2007) focused on different factors which might influence results obtained with this method. The authors emphasize that instructions of whether to report the onset or end of the own movement influence participants’ estimations. They also suggest that the luminance of the clock hand and its size might have an influence on the effects found. Additionally, tasks employing the Libet Clock are visually demanding, as participants have to follow the clock hand with their eyes to make accurate temporal judgments. Thus, the setup is ill-suited for many conceivable settings, especially when they involve tasks with visual effects.

To reduce the task’s inherent visual load and to introduce more flexibility in the experimental task, Cornelio Martinez, Maggioni, Hornbæk, Obrist, and Subramanian (2018) proposed an “auditory Libet Clock.” This method uses spoken letters, which are presented over headphones, rather than the visual clock hand to determine the perceived timing of the actions or events. To the best of our knowledge, at this point, this is still the first study using an auditory timer to measure temporal binding, and the obtained results remain to be replicated and extended. Thus, a thorough and reliable approach to systematically studying temporal binding by means of an auditory timer is needed. The seemingly trivial setup of timed auditory cues has various obvious and less obvious design factors that might affect experimental results and the overall aptness of the method. In this line of research, we varied four design factors that we consider most important and substantial for the design of an auditory measure for temporal binding. Therefore, we systematically manipulated the factors *interval length*, *interval filling*, *sequence predictability*, and *sequence length* of an auditory timer to study temporal binding in a task with visual effects.

First, interval length, which is the length (duration) of the presented letters, is of utmost importance, as it determines the temporal resolution of the timed auditory stimuli. The shorter the interval, the higher the resolution; however, this resolution gain can come at the cost of discernibility of the individual letters. Hence, we ask: *What is the optimal interval length?*

Second, interval filling also plays an important role in the configuration of an auditory timer, as it contributes to its temporal resolution. Additionally, it provides anchors for temporal estimations. Previously and subsequently used letters can be used as temporal cues and therefore serve as anchors for participants’ estimations. The salience of these anchors varies with the filling of the interval. Finally, filling time intervals with auditory stimulation can potentially increase the accuracy of duration estimation (Rammsayer & Lima, 1991). Thus, we seek to answer the question: *How should intervals be filled?*

Third, the predictability of the letter sequence appears to be an important factor, as it might influence participants’ estimation strategies. With decreasing sequence predictability, participants might focus more on auditory anchors while relying less on strategies (e.g., always acting on the same auditory cue). Thus, we ask: *Should the sequence of auditory cues be predictable?*

Ultimately, the number of letters that constitute the auditory scale most likely has an influence on participants’ task load. With increasing length of the letter sequence, it should become more difficult to remember it and therefore draw more cognitive resources. Therefore, we aim to answer the question: *What is the optimal number of auditory cues?*

The presented experiments introduce a thorough, theory-driven approach to establishing an auditory timer for measuring temporal binding. Within this context, the four aforementioned factors are systematically manipulated in successive experiments to find the most suitable timing configuration. All experiments were preregistered on the Open Science Framework (OSF) and were approved by the ethics committee of the psychology department of the Julius-Maximilians-University of Würzburg (GZ 2019-09). All raw data and analysis scripts are available at the project repository (https://osf.io/d3vz5/).

## Experiment 1: Manipulation of interval length

Experiment 1 tested for the ideal presentation length of letters that constitute the auditory timer for measuring temporal binding. This is what we will refer to as interval length. Letters were either 250 ms, 500 ms, or 750 ms long (for more detail see Apparatus and stimuli). According to the study by Cornelio Martinez et al. (2018), we expected to find temporal binding in the 250 ms condition. Additionally, we were interested to find out how variations in the interval length influence temporal binding as an objective measure. As a manipulation check, both action binding and effect binding should be similar to both types of binding found in previous studies using the Libet Clock. Additionally, we collected participants’ perceived task load in order to determine whether there were differences in the subjective quality of the auditory timer depending on the interval lengths.

### Methods

#### Participants

Forty-eight participants (11 male, 8 left-handed, mean age = 24.1 years, *SD* = 6.3) recruited over the university’s participant pool (SONA) took part in the experiment. Prior to data collection, a power analysis for paired-sample *t*-tests was performed using G*Power 3.1 (Faul, Erdfelder, Buchner, & Lang, 2009). Because previous studies have found medium effect sizes for action binding (e.g., Ruess, Thomaschke, & Kiesel, 2017b), we conducted the power analysis with *d* = 0.40, α = .05. With these parameters, a sample size of 41 would have sufficed to ensure high power (.80). However, in order to counterbalance the conditions, we set the sample size to 48. Prior to the experiment, participants signed an informed consent form and they received either monetary compensation or partial course credit for their voluntary participation. All participants were naïve to the purpose of the study and were debriefed afterwards.

#### Apparatus and stimuli

##### Visual effect task

**Fig. 1:**
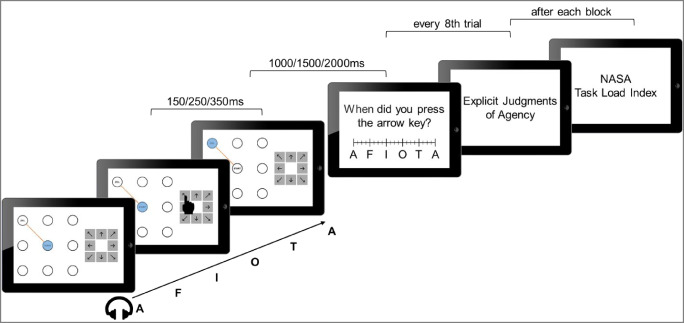
Trial procedure in the experiments. The figure shows an example for a trial in the action experimental condition. Participants saw a 3 × 3 grid of circles on the left side of the screen and were asked to perform keypresses according to the directions given by the indicated goal area. During the trials they heard a sequence of German letters over headphones, which were subsequently used to report the timing of either the keypress or the cursor movement. After every eighth trial, participants had to answer three questions to give explicit agency ratings. Finally, participants completed the NASA Task Load Index (Hart & Staveland, 1988) at the end of each block

The visual effect task was a single-choice task with a visual effect, i.e., the movement of a cursor. It was completed on an iPad 2, which participants operated with the index finger of their right hand. The iPad’s LED screen, with a 9.7-inch diagonal and a resolution of 1024 × 768 px, was used in landscape mode. Compared to normal keyboards, a touch device gives the user more unambiguous feedback as to when the finger touched the surface. In contrast, with a standard keyboard, there are at least two events that might shape the experienced point in time of that keypress, namely when the finger hit the key and when the key was completely pressed. Additionally, this addresses the pitfalls inherent in other sensory input such as clicking sounds elicited by the keypress that usually accompany the use of computer keyboards. Thus, touchscreen devices seem to be suitable for studying temporal binding^1^. During the experiment, a 3 × 3 grid of circles with diameters of 100 px was presented on the left half of the screen (see Fig. 1). Next to the grid on the right was a keypad with eight spatially arranged arrow keys, each of which measured 100 × 100 px. At trial onset, the center circle (start area) was filled in blue (to illustrate a movable cursor) and displayed the German word for start (“Start”). Simultaneously, one of the other eight circles in the grid displayed the German word for goal (“Ziel”) and was connected to the start area with a straight orange line. The goal location indicated which keypress participants had to perform.

##### Auditory timer task

During trials, participants repeatedly heard five timed letters over headphones at a preset volume. This letter sequence, consisting of the German letters A, F, I, O, and T, served as auditory timer to reference the perceived timing of actions and effects. In the first experiment, we decided to use a sequence of five letters to ensure that participants would be able to store the entire sequence in their working memory while executing the visual task. Moreover, the selected number of auditory stimuli provided a good temporal resolution when transferred to the visual scale on the iPads, where one pixel represented 2.5 ms (for a systematic manipulation of the number of letters, see Experiment 4: Manipulation of sequence length). The timed auditory letter sequence was designed so that the offset of one letter constituted the onset of the next, so there was no pause in between. In Experiment 1, we varied the length of each letter on three levels^2^ (250 ms, 500 ms, 750 ms) between blocks. This resulted in continuous streams of letters that varied only in the broadness of the pronunciation. A representative example of the auditory stream is accessible at the project’s OSF page (https://osf.io/2746f/).

#### Procedure

**Fig. 2:**
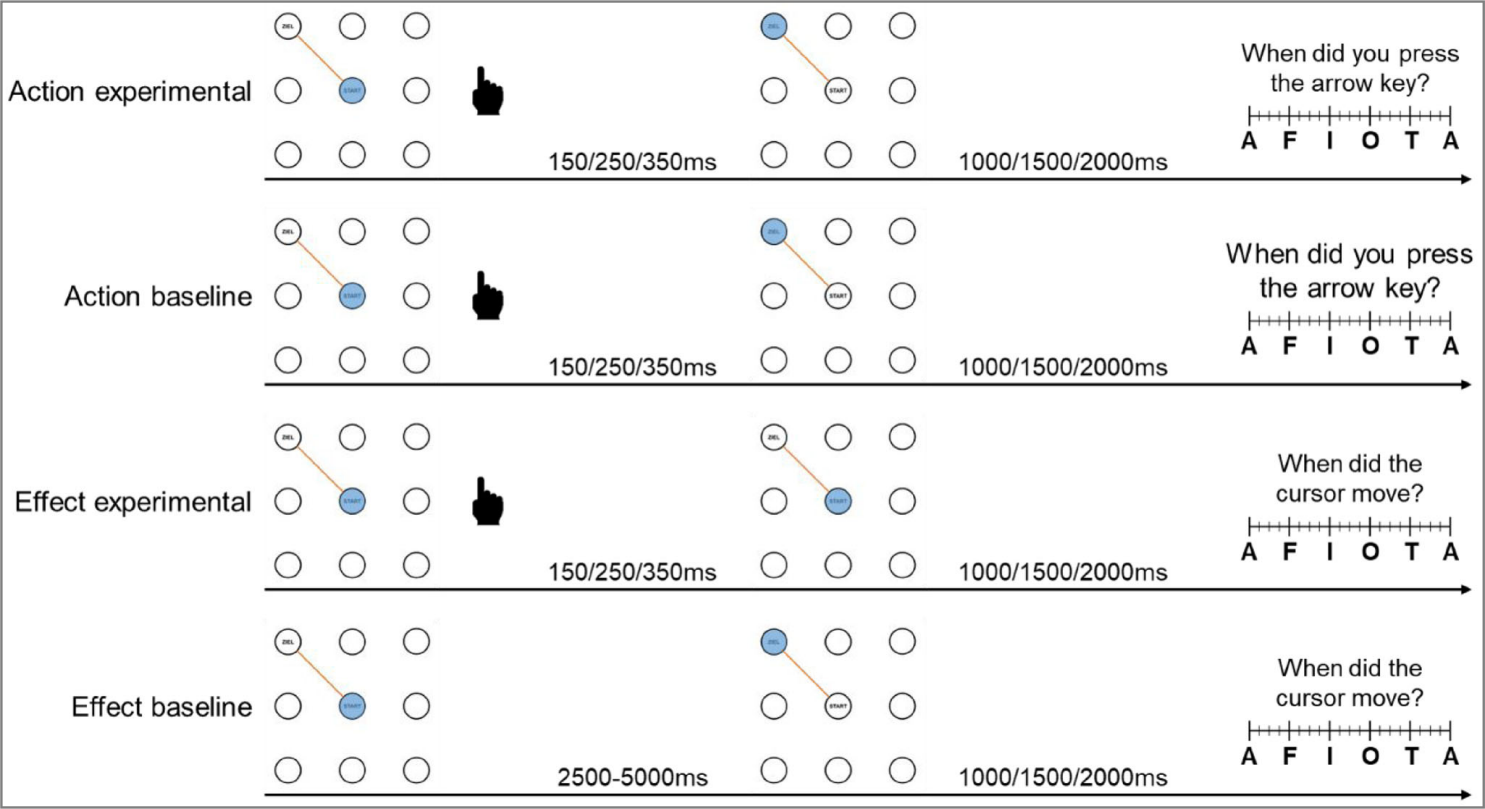
Conditions. (1) In the action experimental condition, participants' keypresses make the cursor move. Subsequently, participants report the timing of their keypress on the scale. (2) In the action baseline condition, participants again press an arrow key. However, this time it does not cause the cursor to move; rather, the cursor stays at the same position after a delay of 150, 250 or 350 ms. Afterwards, participants report the timing of their keypress. (3) In the effect experimental condition, participants’ keypresses cause the cursor to move. At the end of the trial, participants are asked to report the timing of the cursor movement. (4) In the effect baseline condition, participants do not perform a keypress. However, after a random delay of 2500–5000 ms, the cursor moves from the start area into the goal area. Subsequently, participants report the timing of the cursor movement

Participants encountered four different estimation conditions throughout the experiment (see Fig. 2): (1) Action experimental: Cursor movements followed participants’ keypresses and the perceived timing of the keypress was assessed. (2) Action baseline: Participants’ keypresses were not followed by a cursor movement and the perceived timing of the keypress was assessed. (3) Effect experimental: Cursor movements followed participants’ keypresses and the perceived timing of the cursor movement was assessed. (4) Effect baseline: After a random interval of 2500–5000 ms, a cursor movement occurred without participants’ keypresses and the perceived timing of this cursor movement was assessed. These conditions were used to calculate temporal binding (see Results for more detail). As temporal binding is calculated as the difference between participants’ estimation errors in the experimental compared to the baseline condition, absolute estimation errors will not be reported here, but can be retrieved from the OSF repository (https://osf.io/d3vz5/).

At trial onset, participants saw the grid on the left side of the screen and the keypad on the right side while hearing the letter sequence. The first letter of the letter sequence was selected at random. The circle in the middle of the grid was colored in blue and displayed the German word for start. Simultaneously, one of the other eight circles showed the German word for goal. These two circles were connected with a straight orange line, informing participants which key to press. Participants were asked to press the corresponding arrow key to move the cursor from the start area to the goal area. Additionally, participants received the instruction to wait at least three letters until they performed the keypress. They were also discouraged from pre-planning the time of their keypress and received the explicit information that this was not a speed task, but rather that they could perform the keypresses at their leisure.

In the experimental conditions, their keypress was followed by the respective cursor movement after a random delay of 150, 250, or 350 ms. These delays were chosen in accordance with previous studies (e.g., Haggard et al., 2002; Ruess, Thomaschke, & Kiesel, 2017b; Weller, Schwarz, Kunde, & Pfister, 2020). We used varying delays so participants could not compute the timing of their action by simply subtracting a fixed interval from the perceived timing of the effect and vice versa. This way, they had to concentrate more intently on the event in question. In the action baseline condition, participants only performed a keypress which did not cause the cursor to move. In the effect baseline condition, participants were asked not to press a key. In this condition, the cursor moved after a random delay of 2500–5000 ms after trial onset.

After the last event in each condition (i.e., cursor movement in the experimental conditions and effect baseline condition; keypress in the action baseline condition), the spoken letters presented over the headphones continued for another 1000, 1500, or 2000 ms. Subsequently, participants were asked to report the perceived timing of either their action or the cursor movement by locating it on a visual scale displaying the letter sequence (A-F-I-O-T-A), with the first and last letter being the same to ensure that the entire range of possible estimations was covered. The scale was presented in the center of the screen with a width of 1000 px and a height of 100 px. It had six anchors for each letter, which had three subdivisions each (see Fig. 2). Participants could press any point on the scale to make their temporal judgment. Subsequently, this was translated into a continuous dependent variable reflecting participants’ temporal estimation, 1 px = 2.5 ms, for further analyses. Following correct responses, the next trial started, with an inter-trial interval of 2000 ms, with the presentation of the grid, the start and a new goal area, and the keypad. In cases where participants’ keypresses did not correspond to the predefined path, the cursor followed participants’ keypresses rather than the orange line, and an error message was displayed. After such commission errors, participants received an error message in the form of the German word for error (“Fehler”) in red font in the center of the circle grid. If participants pressed a key in the effect baseline condition, they were informed not to press a button in the same way. This feedback was displayed after the cursor movement was completed and before participants had to give their time estimations.

In addition to the perceived timing, participants made explicit agency judgments on a continuous 100-point scale from −50 to 50. Participants rated their perceived authorship (“The dot moved as I wanted it to”), control (“I controlled the dot’s movement”), and causation (“I caused the dot’s movement”) over the cursor movement. These ratings were given after every eighth trial in the experimental blocks.

As the variable of interest for this experiment was the interval length, this factor was manipulated within subjects. For counterbalancing, we divided the experiment into thirds and assigned a specific interval length (250, 500, or 750 ms) to each of them. The sequence of the four estimation conditions was also counterbalanced across participants, with the prerequisite that they always had to start with the baseline blocks before completing the experimental blocks. The sequence of conditions remained the same throughout all experimental thirds. Overall, participants completed 12 blocks (two baseline blocks, then two experimental blocks, for every interval length) of 40 trials each.

At the end of each third, participants filled out a German version of the NASA Task Load Index (TLX) consisting of six items to investigate subjective task load (Hart & Staveland, 1988). It assesses mental demand, physical demand, temporal demand, performance, effort, and frustration on a continuous 10-point scale from low to high. The experiment took about 90 minutes.

Raw data and analysis scripts are available on the Open Science Framework, https://osf.io/d3vz5/.

#### Design

The study used a 3 × 4 repeated-measures design with interval length (250 ms vs. 500 ms vs. 750 ms) and condition (action experimental vs. action baseline vs. effect experimental vs. effect baseline) as within-subjects factors.

#### Data analysis

To assess temporal binding, we first calculated estimation errors as the difference between participants’ temporal estimates and the actual timing of the respective event (timing_estimation_ − timing_actual_). For example, if participants pressed a key 100 ms after they heard the letter “I” but reported this key press as having occurred in the middle between “I” and “O” (i.e., 250 ms after the onset of letter “I”), the estimation error for this particular trial was (250 ms − 100 ms) 150 ms. We discarded erroneous trials and trials in which the temporal binding exceeded 2.5 SDs of the participant’s cell mean in the respective condition (baseline vs. experimental; 250 ms vs. 500 ms vs. 750 ms). Subsequently, we calculated means for each estimation condition and interval length separately. These were then used to calculate the action binding and the effect binding for each interval length. Therefore, participants’ estimation errors in the baseline conditions were subtracted from those in the respective experimental conditions (temporal binding = estimation error_exp_ − estimation error_base_). Positive values indicate that an occurrence in the experimental condition was perceived to have happened later than in the baseline condition, while negative values indicate an earlier perception of an occurrence in the experimental compared to the baseline condition.

To test our hypothesis, we first conducted separate two-tailed *t*-tests for all types of action binding and effect binding to see whether the differences between experimental and baseline conditions differed significantly from zero, that is, whether participants showed temporal binding. Then, we conducted two one-factorial analyses of variance (ANOVAs), one for action binding and one for effect binding, with interval length (250, 500, 750 ms) as within-subjects factor to uncover specific differences between the individual interval lengths. Follow-up analyses were conducted via two-tailed, paired *t*-tests. Effect sizes for all paired *t*-tests were calculated as *d*_*z*_ = $\frac{t}{\sqrt{n}}$.

For explicit agency judgments, we calculated mean scores for explicit agency ratings (authorship, control, causation) for each condition (action experimental, effect experimental) and each interval length individually. Then, a one-way ANOVA with condition (action vs. effect) as within-subjects factor was conducted to uncover differences in participants’ subjective judgments of agency between conditions in which participants focused either on the action or on the effect. Ultimately, three repeated-measures ANOVAs with interval length (250 ms vs. 500 ms vs. 750 ms) as within-subjects factor were conducted.

To assess participants’ task load with different interval lengths, mean scores for each scale of the NASA TLX were calculated and compared between the three interval lengths. A repeated-measures ANOVA with interval length (250 ms vs. 500 ms. vs. 750 ms) as within-subjects factor was conducted separately for each scale. Follow-up analyses were carried out via two-tailed, paired *t*-tests. Effect sizes for all paired *t*-tests were calculated as *d*_*z*_ = $\frac{t}{\sqrt{n}}$.

Additionally, for nonsignificant results, we used post-hoc Bayes analyses to further examine the evidence for and against the null hypothesis. We calculated Bayes factors using JASP computer software (JASP Team, 2018). As stated in the preregistration, we expected medium to large effects. Thus, we used a scale parameter of 0.25 for the analyses. This corresponds to a probability of 80% that the effect lies between −0.8 and 0.8. As per convention, a Bayes factor of *BF*_*10*_ < 1/3 can be interpreted as evidence in favor of the null hypothesis, while Bayes factors (*BF*_*10*_) greater than 3 yield at least moderate evidence for the alternative hypothesis (Dienes, 2014). As we tested for equality, however, we used the inverse *BF*_*01*_ (with ${BF}_{01}=\frac{1}{B{F}_{10}}\Big)$ and thus the inverse decision criteria apply (see also Janczyk & Pfister, 2020).

### Results

#### Temporal binding

Erroneous trials (0.8%) and trials in which temporal binding exceeded 2.5 SDs of the participant’s cell mean (2.6%) were excluded from the analyses. Errors occurred mainly in the first trials of effect baseline blocks in which participants were asked not to press a key. Nevertheless, error rates showed obvious floor effects. Therefore, error rates will not be analyzed further (see Dixon, 2008 for comments regarding floor and ceiling effects in the analysis of error data).

##### Action binding

Data showed significantly larger estimation errors for experimental conditions than for baseline conditions for all comparisons except the action binding in the 750 ms condition, *t*_*250*_(47) = 2.57, *p* = .013, *d*_*z*_ = 0.37, ∆ = 23.06 ms, *t*_*500*_(47) = 4.10, *p* < .001, *d*_*z*_ = 0.59, ∆ = 51.89 ms, *t*_*750*_(47) = 1.46, *p* = .151, *d*_*z*_ = 0.21, ∆ = 39.22 ms. That is, the action was overall reported to be shifted towards the effect, while this was not the case in the 750 ms condition. Participants did indeed judge actions to have occurred later in time when they were followed by a cursor movement than when they were executed in isolation.

**Fig. 3:**
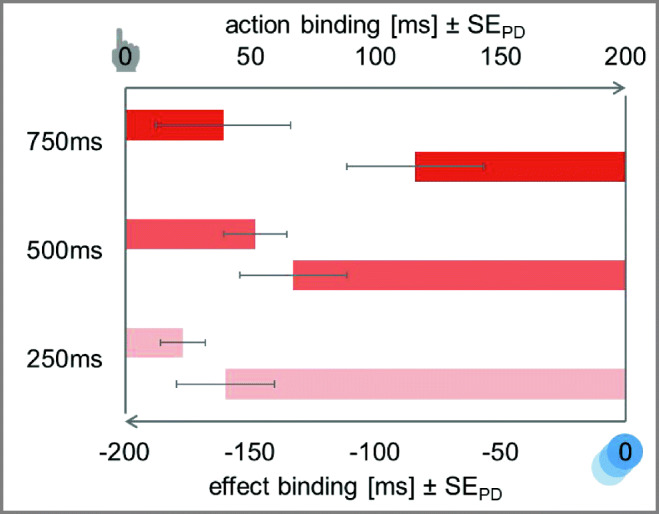
Temporal binding in Experiment 1. Action binding and effect binding relative to the baseline condition. The *y*-axis intercept denotes the perceived timing of the action (top) and the perceived timing of the effect (bottom) in the respective baseline conditions. Action binding is shown as bars from left to right to indicate the perceived delay of the action. Effect binding is shown as bars from right to left to indicate the perceived advancement of the effect. Error bars depict standard errors of paired differences for the factor interval length (Pfister & Janczyk, 2013)

The ANOVA for action binding with interval length (250 ms vs. 500 ms vs. 750 ms) as within-subjects factor did not show any significant difference in the magnitude of action binding between the three interval lengths, *F* < 1, *BF*_*01*_ = 7.71 (see Fig. 3).

##### Effect binding

Estimation errors of effect differed significantly between experimental and baseline conditions for all three interval lengths, *t*_*250*_(47) = −8.21, *p* < .001, *d*_*z*_ = 1.18, ∆ = −159.77 ms, *t*_*500*_(47) = −6.26, *p* < .001, *d*_*z*_ = 0.90, ∆ = −132.74 ms, *t*_*750*_(47) = −3.08, *p* = .003, *d*_*z*_ = 0.44, ∆ = −83.97 ms. Cursor movements were reported to have happened earlier when a keypress preceded this cursor movement.

The ANOVA for effect binding with interval length (250 ms vs. 500 ms vs. 750 ms) as within-subjects factor revealed a significant difference in binding size between the different interval lengths, *F*(2,94) = 5.15, *p* = .008, η_p_^2^ = .10. That is, effect binding increased significantly between the 750 ms and the 250 ms condition, *t*(47) = −2.79, *p* = .008, *d*_*z*_ = 0.40, and between the 750 ms and the 500 ms condition, *t*(47) = −2.08, *p* = .043, *d*_*z*_ = 0.30. There was no clear evidence for or against a difference between the short and medium interval length, *t*(47) = −1.28, *p* = .206, *d*_*z*_ = 0.18, *BF*_*01*_ = 1.49.

#### Explicit agency judgments

Explicit judgments of agency did not differ between conditions (i.e., action experimental vs. effect experimental), *F*(1,47) = 1.25, *p* = .270, η_p_^2^ = .03, *BF*_*01*_ = 9.38, so explicit agency judgments were calculated across conditions. In general, agency ratings were high for all three types of judgment, authorship (*M* = 25.23, *SD* = 19.54), control (*M* = 22.59, *SD* = 20.72), and causation (*M* = 35.17, *SD* = 13.85).

**Fig. 4:**
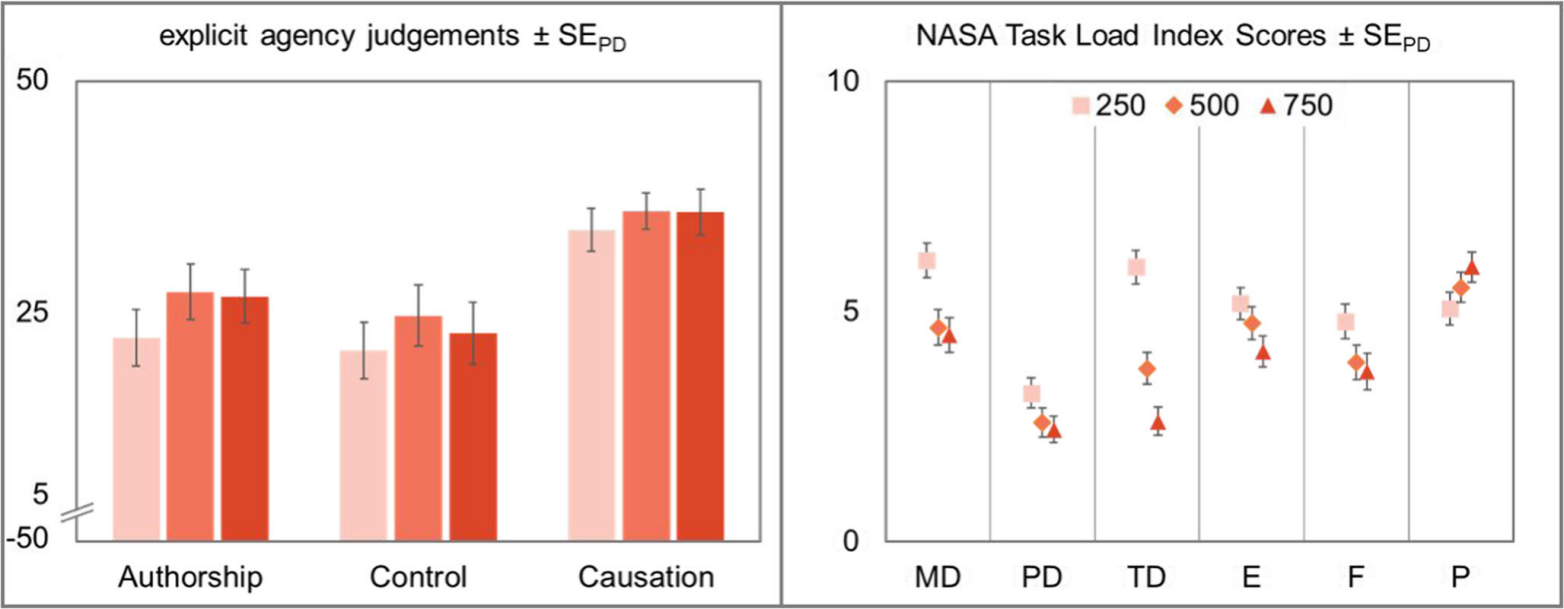
Left: Explicit agency judgments for authorship, control, and causation of the cursor movement. Agency judgments were made on a scale from −50 to 50 after every eighth trial in all experimental conditions. Right: Perceived task load as measured with the NASA Task Load Index (Hart & Staveland, 1988). MD: mental demand, PD: physical demand, TD: temporal demand, E: effort, F: frustration, P: performance. Squares represent participants’ judgments with letters of 250 ms, diamonds 500 ms, and triangles letters with a length of 750 ms. Error bars in both panels depict standard errors of paired differences for the factor interval length (Pfister & Janczyk, 2013)

Subsequently, three repeated-measures ANOVAs with interval length (250 ms vs. 500 ms vs. 750 ms) as within-subjects factor were conducted. Explicit authorship ratings differed significantly between the different interval lengths, *F*(2,94) = 4.75, *p* = .011, η_p_^2^ = .09. This effect was mainly due to participants’ significantly lower authorship ratings in the 250 ms condition compared to the 500 ms condition, *t*(47) = −2.73, *p* = .009, *d*_*z*_ = −0.39, while their ratings in the 500 ms and the 750 ms condition did not show clear evidence for or against a difference, *t* < 1, *BF*_*01*_ = 2.58. Explicit agency judgments for control and causation were not influenced by interval length, *F*_*control*_(2,94) = 1.51, *p* = .226, η_p_^2^ = .03, *BF*_*01*_ = 4.18, *F*_*causation*_ < 1, *BF*_*01*_ = 7.41 (see Fig. 4).

#### NASA Task Load Index

Participants filled out the NASA Task Load Index to determine whether the manipulation of interval length had an effect on perceived task load. Here we report only the subscales on which interval length had an influence. All other results can be found on the OSF repository (https://osf.io/d3vz5/).

Data showed a significant effect of interval length on mental demand (MD), *F*(2,94) = 16.19, *p* < .001, η_p_^2^ = .26. Mental demand decreased significantly between the 250 ms and the 500 ms condition, *t*(47) = 4.61, *p* < .001, *d*_*z*_ = 0.67, and between the 250 ms and the 750 ms condition, *t*(47) = 5.56, *p* < .001, *d*_*z*_ = 0.80, while there was no clear evidence for or against a difference between the two longer intervals, *t* < 1, *d*_*z*_ = 0.07, *BF*_*01*_ = 2.45.

The same held true for physical demand (PD). It differed significantly between the three interval lengths, *F*(2,94) = 5.24, *p* = .007, η_p_^2^ = .10. While there was a slight decrease in physical demand between the 250 ms and the 500 ms condition, *t*(47) = 2.11, *p* = .040, *d*_*z*_ = 0.30, and between the 250 ms condition and the 750 ms condition, *t*(47) = 4.71, *p* < .001, *d*_*z*_ = 0.68, there was no clear evidence for or against a difference between the medium and the long interval, *t* < 1, *d*_*z*_ = 0.08, *BF*_*01*_ = 2.39.

The ANOVA for temporal demand (TD) revealed significant differences between the three conditions, *F*(2,94) = 37.04, *p* < .001, η_p_^2^ = .44. Temporal demand decreased significantly from the 250 ms to the 500 ms condition, *t*(47) = 5.59, *p* < .001, *d*_*z*_ = 0.81, as well as from the 250 ms to the 750 ms condition, *t*(47) = 7.80, *p* < .001, *d*_*z*_ = 1.13. Temporal demand in the 500 ms condition was also significantly higher than in the 750 ms condition, *t*(47) = 3.20, *p* = .003, *d*_*z*_ = 0.46.

Data showed a significant effect of interval length on performance (P), *F*(2,94) = 4.48, *p* = .014, η_p_^2^ = .09. Performance gradually increased with increasing interval length. However, there was neither evidence for nor against a difference between either the 250 ms and the 500 ms condition, *t*(47) = −1.60, *p* = .115, *d*_*z*_ = 0.23, *BF*_*01*_ = 1.07, or the 500 ms and the 750 ms condition, *t*(47) = −1.39, *p* = .170, *d*_*z*_ = 0.20, *BF*_*01*_ = 1.34. Performance in the 250 ms condition was rated significantly higher than in the 750 ms condition, *t*(47) = −3.02, *p* = .004, *d*_*z*_ = 0.44.

Data showed a significant effect of interval length on effort (E), *F*(2,94) = 4.36, *p* = .016, η_p_^2^ = .09. Effort gradually decreased with increasing interval length. Effort was significantly lower in the 750 ms condition than in the 250 ms condition, *t*(47) = 3.00, *p* = .004, *d*_*z*_ = 0.43. Further analyses did not show any clear evidence for or against a difference between the 250 ms condition and the 500 ms condition, *t*(47) = 1.15, *p* = .258, *d*_*z*_ = 0.17, *BF*_*01*_ = 1.67, or between the 500 ms condition and the 750 ms condition, *t*(47) = 1.78, *p* = .081, *d*_*z*_ = 0.26, *BF*_*01*_ = 0.86.

The ANOVA revealed a significant effect of interval length on frustration (F), *F*(2,94) = 4.31, *p* = .016, η_p_^2^ = .08. Frustration decreased significantly from the 250 ms and the 500 ms condition, *t*(47) = 2.58, *p* = .013, *d*_*z*_ = 0.37, and from the 250 ms condition to the 750 ms condition, *t*(47) = 2.58, *p* = .013, *d*_*z*_ = 0.37, while there was no clear evidence for or against a difference between the two longer intervals, *t* < 1, *BF*_*01*_ = 2.46.

### Discussion

We investigated whether varying lengths of the letters constituting the auditory timer have an influence on temporal binding. Experiment 1 served the purpose of determining the optimal interval length for our setup. Participants executed a navigation task on an iPad while hearing timed auditory stimuli over headphones. These stimuli were five German letters with three different interval lengths (250, 500, 750 ms). All interval lengths produced effect binding, and the perceived timing of actions in all conditions tended to be shifted towards the effect. However, action binding did not differ significantly from zero in the condition with letters of 750 ms. These results are in line with previous studies using temporal binding as a measure, which also report smaller action binding than effect binding (Beck, Di Costa, & Haggard, 2017; Ruess, Thomaschke, & Kiesel, 2017b). Thus, we conclude that our setup is in principle capable of measuring temporal binding and of replicating previous findings on temporal binding.

All interval lengths showed medium to large effects for effect binding. This, as well as the absolute magnitude of the estimation errors, replicates previous studies examining temporal binding by means of a visual Libet Clock (Ruess, Thomaschke, & Kiesel, 2017b; Schwarz, Weller, Klaffehn, & Pfister, 2019a; Wolpe, Haggard, Siebner, & Rowe, 2013). As effect binding did not differ significantly between short and medium intervals, it seems that there is not one ideal interval length for measuring temporal binding with an auditory timer. Rather, it appears that auditory stimuli with short to medium length, remaining below a certain threshold (in this case 750 ms), seem to be suitable for revealing temporal binding. The same applies for action binding; both effect sizes and absolute estimation errors replicated previous studies at least for the two shorter interval lengths. Therefore, our recommendation is that the auditory stimuli be no shorter than 250 ms but not longer than 500 ms.

Contrary to the implicit temporal binding measures, the length of the presented auditory stimuli did not influence explicit agency judgments. Throughout the experiment, participants rated their sense of agency as high in almost all conditions. The only condition in which explicit sense of agency was slightly diminished was when participants had to rate their authorship over the cursor movements in the 250 ms condition. Previous studies with predictable action–outcome delays have shown that increasing these delays (>200 ms) produces lower explicit agency ratings (Wen, Yamashita, & Asama, 2015). In the present study, action–outcome delays varied on a trial-by-trial basis between 150 ms and 350 ms. Additionally, agency ratings were recorded after every eighth trial, rendering it impossible to map agency ratings to specific action–outcome delays. Therefore, it is plausible that participants made an overall judgment across the previous mini-block, resulting in less differentiated judgments of agency. To sum up, interval length does not seem to have a great influence on participants’ explicit agency judgments, which can therefore be neglected when designing the auditory timer. Researchers should however also bear in mind participants’ task load and frustration during task execution, as this is often detrimental to their concentration and task irrelevant thoughts over the course of the experimental session.

Over the course of the experiment, there was a trend that task load decreased with increasing interval lengths. This was also the case for participants’ perceived effort and frustration, which decreased as the length of the presented letters increased. This pattern reversed for participants’ self-ratings of performance. They judged themselves as doing better on task completion when interval length increased. Consequently, we recommend the utilization of intervals with a medium length for the auditory timer. This way, researches can ensure low to moderate task load while also maintaining participants’ self-image as being competent on the task.

To sum up, with regard to the temporal estimation measure, we decided to use an interval length of 500 ms for subsequent studies. This interval length appeared to create the most robust action binding while also producing reasonably large effect binding. Additionally, considering participants’ task load ratings, the 500 ms interval seemed to evoke a tolerable task load, whereas even shorter intervals unnecessarily increased task load and at the same time descriptively lowered subjective performance ratings. This design decision is supported by participants’ explicit agency judgments, which tended to be slightly lower in the 250 ms condition than in the 500 ms condition.

## Experiment 2: Manipulation of interval filling

In Experiment 2 we systematically manipulated the factor interval filling, that is, the way in which the spoken letters were presented. This design factor was chosen as it contributes to the temporal resolution of the auditory timer. Letters were presented in three different ways: filled, half-filled, and sequenced. We expected half-filled intervals to be a poor measure for temporal binding, as the silence in the second half of the interval does not provide temporal information. On the contrary, sequenced intervals should provide participants with more anchors and therefore make temporal judgments easier. The addition of temporal information should however also lead to increased task load.

### Methods

#### Participants

A new set of 48 participants (15 male, 4 left-handed) with a mean age of 28.42 years (*SD* = 9.70) were recruited and fulfilled the same criteria as in Experiment 1.

#### Apparatus and stimuli

**Fig. 5:**
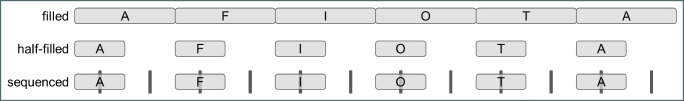
Manipulation of the interval filling in Experiment 2. In the filled condition, letters were 500 ms long, and the offset of one letter marked the onset of the next. In the half-filled condition, spoken letters were 250 ms long and were followed by 250 ms of silence before the onset of the next letter. The sequenced condition consisted of spoken letters of 250 ms and a 250 ms pause. Additionally, metronome-like clicks (depicted here by the dark lines) were presented after 125 ms and 375 ms in order to aid participants’ temporal resolution. Representative examples can be found on the project’s OSF page (https://osf.io/d3vz5/)

The visual task was left unchanged from Experiment 1. For the auditory timer, participants again heard the German letters A, F, I, O, and T over headphones. But this time we varied the filling of the letter intervals on three levels (filled, half-filled, or sequenced) between blocks. In the filled condition, the entire 500 ms interval was filled with a spoken letter. In the half-filled condition, intervals consisted of spoken letters (250 ms) followed by 250 ms of silence until the end of the interval. In the sequenced condition, there was a steady metronome-like timer consisting of short clicks with a speed of four clicks per second. This timer was synchronized with the spoken letters such that there was a click in the middle of the spoken letter (at 125 ms) and one click halfway through the silence following the letter, that is, at 375 ms after the letter onset. Figure 5 shows the three different interval fillings.

#### Procedure

The procedure for Experiment 2 followed that for Experiment 1. As the variable of interest in Experiment 2 was the interval filling, this factor was manipulated within subjects, and we divided the experiment into thirds and assigned a specific interval filling (filled, half-filled, or sequenced) to each third. The order of interval fillings was counterbalanced across participants.

#### Design

The study used a 3 × 4 repeated-measures design with interval filling (filled vs. half-filled vs. sequenced) and condition (action experimental vs. action baseline vs. effect experimental vs. effect baseline) as within-subjects factors.

### Results

Data analysis in Experiment 2 followed that described in Experiment 1.

#### Temporal binding

Erroneous trials (0.8%) and outliers, trials in which temporal binding exceeded 2.5 SDs of the participant’s cell mean (2.7%), were excluded from the analyses.

##### Action binding

**Fig. 6:**
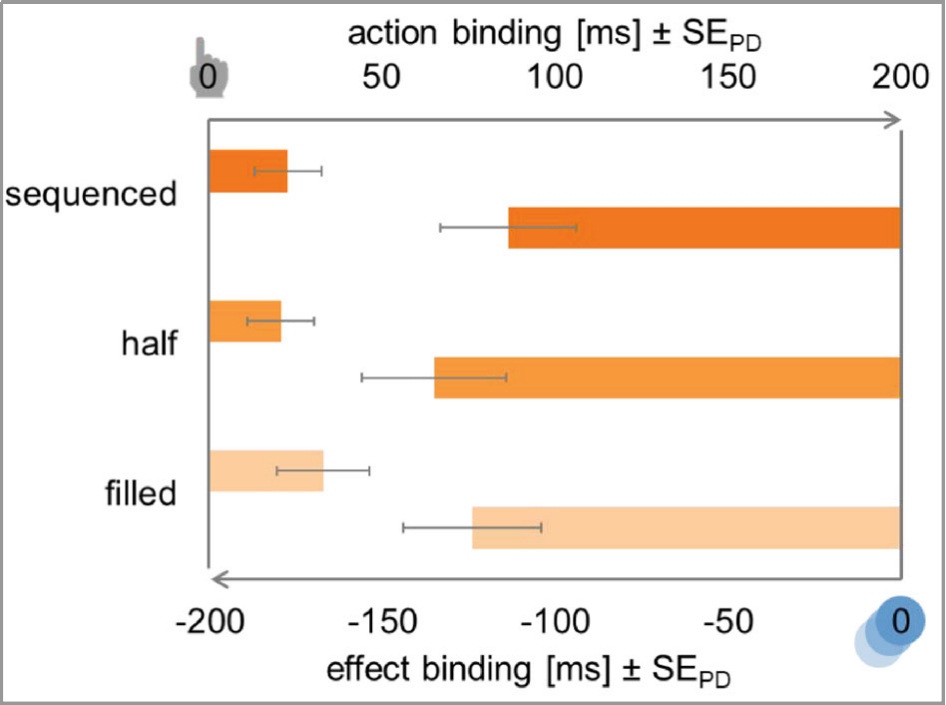
Temporal binding in Experiment 2. Action binding and effect binding relative to the baseline condition. The *y*-axis intercept denotes the perceived timing of the action (top) and the perceived timing of the effect (bottom) in the respective baseline conditions. Action binding is shown as bars from left to right to indicate the perceived delay of the action. Effect binding is shown as bars from right to left to indicate the perceived advancement of the effect. Error bars depict standard errors of paired differences for the factor interval filling (Pfister & Janczyk, 2013)

Participants showed action binding irrespective of the interval filling. That is, actions were perceived to have happened later in the filled condition, *t*(47) = 2.48, *p* = .017, *d*_*z*_ = 0.36, ∆ = 33.27 ms, as well as the half-filled condition, *t*(47) = 2.19, *p* = .033, *d*_*z*_ = 0.32, ∆ = 20.95 ms, and the sequenced condition, *t*(47) = 2.41, *p* = .020, *d*_*z*_ = 0.35, ∆ = 23.07 ms. Participants did indeed judge actions to have occurred later in time when they were followed by a cursor movement than when they were executed in isolation (see Fig. 6).

The ANOVA for action binding with interval filling (filled vs. half-filled vs. sequenced) as within-subjects factor did not show any significant difference in the magnitude of action binding between the three interval fillings, *F* < 1, *BF*_*01*_ = 10.23.

##### Effect binding

Cursor movements in all three conditions were perceived to be shifted towards the preceding action, *t*_*filled*_(47) = −6.21, *p* < .001, *d*_*z*_ = 0.90, ∆ = −124.10 ms, *t*_*half*_(47) = −6.46, *p* < .001, *d*_*z*_ = 0.93, ∆ = −135.26 ms, *t*_*sequenced*_(47) = −5.77, *p* < .001, *d*_*z*_ = 0.83, ∆ = −113.74 ms. That is, cursor movements were perceived to have happened earlier when a keypress preceded this cursor movement.

The ANOVA for effect binding with interval filling (filled vs. half-filled vs. sequenced) as a within-subjects factor also showed no significant differences between the interval fillings, *F* < 1, *BF*_*01*_ = 9.12.

#### Explicit agency judgments

As in Experiment 1, there was no significant difference in judgments of agency between action experimental and effect experimental conditions, *F*(1,47) = 2.59, *p* = .114, η_p_^2^ = .05, *BF*_*01*_ = 3.10. Thus, explicit agency judgments were calculated across conditions. Again, agency ratings were high for all three types of judgment, authorship (*M* = 28.83, *SD* = 19.14), control (*M* = 28.20, *SD* = 20.75), and causation (*M* = 36.48, *SD* = 15.15).

**Fig. 7:**
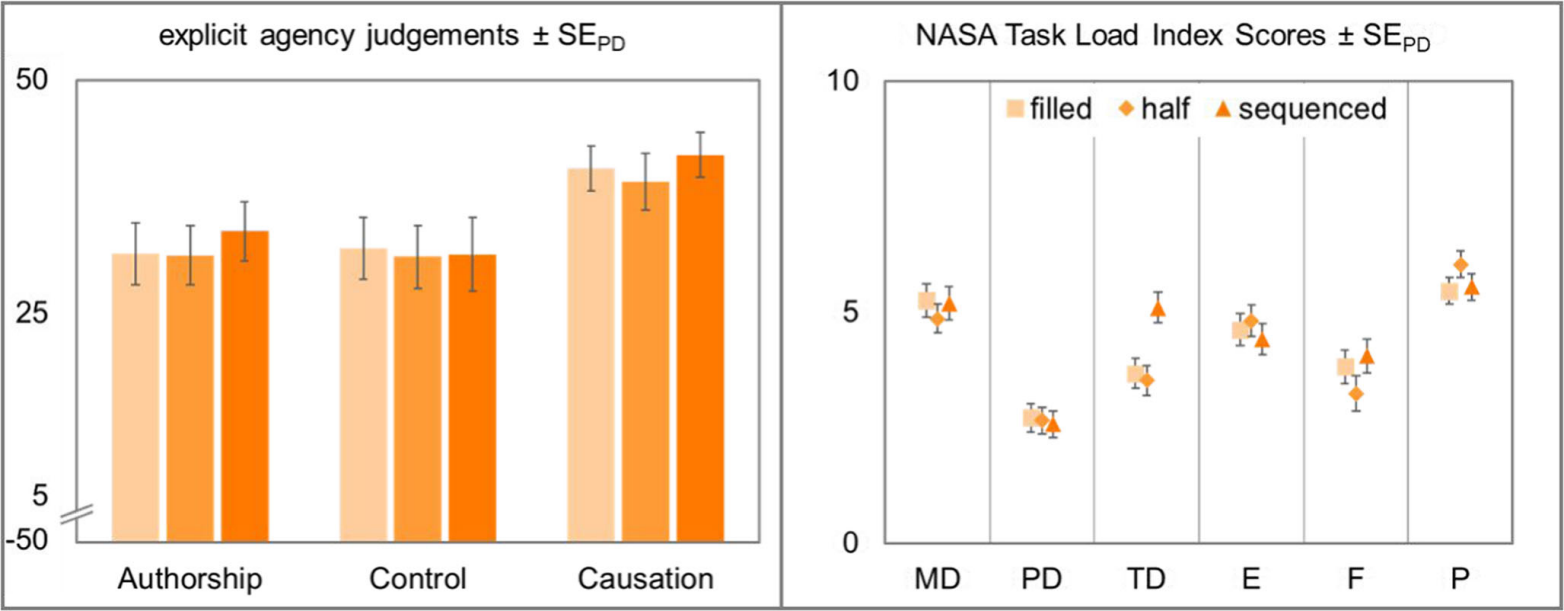
Left: Explicit agency judgments for authorship, control, and causation of the cursor movement. Agency judgments were made on a scale from −50 to 50 after every eighth trial in all experimental conditions. Right: Perceived task load as measured with the NASA Task Load Index (Hart & Staveland, 1988). MD: mental demand, PD: physical demand, TD: temporal demand, E: effort, F: frustration, P: performance. Squares represent participants’ judgments for a sequence with filled letters, diamonds with half-filled letters, and triangles with sequenced letters. All error bars depict standard errors of paired differences for the factor interval length (Pfister & Janczyk, 2013)

Subsequently, three repeated-measures ANOVAs with interval filling (filled vs. half-filled vs. sequenced) as within-subjects factor were conducted. Explicit agency judgments did not differ significantly between the different interval fillings, *F*_*authorship*_(2,94) = 1.14, *p* = .323, η_p_^2^ = .02, *BF*_*01*_ = 5.69, *F*_*control*_ < 1, *BF*_*01*_ = 13.80, *F*_*causation*_(2,94) = 1.13, *p* = .327, η_p_^2^ = .02, *BF*_*01*_ = 5.70 (see Fig. 7).

#### NASA Task Load Index

The ANOVA for temporal demand revealed significant differences between the three interval fillings, *F*(2,94) = 15.01, *p* < .001, η_p_^2^ = .24. Temporal demand did not show clear evidence for or against a difference between filled and half-filled intervals, *t* < 1, *d*_*z*_ = 0.08, *BF*_*01*_ = 2.37. However, temporal demand increased significantly in the sequenced condition compared to the filled letters, *t*(47) = −4.15, *p* < .001, *d*_*z*_ = 0.60, and the half-filled letters, *t*(47) = −4.58, *p* < .001, *d*_*z*_ = 0.66. Interval filling did not have any significant effect on either of the items mental demand, physical demand, or effort, all *F*s < 1, all *BF*_*01*_ > 7.20. Even though there was a descriptive trend towards better performance in the half-filled condition than in the other two, there was neither evidence for nor against any effect of interval filling on performance, *F*(2,94) = 2.50, *p* = .087, η_p_^2^ = .05, *BF*_*01*_ = 1.83. Data also showed no clear evidence for or against an effect of interval filling on frustration, *F*(2,94) = 2.21, *p* = .115, η_p_^2^ = .05, *BF*_*01*_ = 2.29. However, there was a descriptive trend towards lower frustration in the half-filled condition than in the filled and sequenced condition (see Fig. 7).

### Discussion

With Experiment 2, we intended to determine how intervals should be filled. Surprisingly, interval filling influenced neither participants’ temporal estimations nor their task load. That is, contrary to our hypothesis, all interval fillings produced both robust action binding and robust effect binding, which did not differ significantly in size. Again, effect sizes were larger for effect binding, replicating previous results on temporal binding, where effect binding was stronger than action binding (Wolpe et al., 2013).

As attending to the auditory timer is not the primary task, participants’ attention was probably more focused on the visual task than on the design of the auditory timer. This attentional bias might in turn have led to reduced discrimination between the interval fillings. Considering that 250 ms is sufficient to discriminate the letters in our experiments, it is likely that participants simply judged whether the event in question occurred before, after, or during this letter discrimination. What is interesting is that the sequenced filling, which was designed to provide additional time cues, i.e., temporal anchors, also did not influence binding sizes.

On the contrary, sequenced letters increased participants’ perceived task load by leading to higher temporal demand ratings as well as higher frustration when participants judged their performance to be inferior in the sequenced condition.

As explicit agency judgments also did not differ between the three types of interval filling, we conclude that the manipulation does not have strong consequences for our experimental design. Nonetheless, with regard to the NASA TLX, participants seemed to prefer the half-filled letters. This might reflect the fact that this sequence sounded most natural. When we pronounce letter sequences in our daily lives, we usually make short pauses between the letters, akin to the silence in the second half of the half-filled interval. Thus, we decided to use half-filled letters for subsequent studies. It is however worth noting that researchers may adjust the filling according to their needs and stimuli without risk of sabotaging their data.

## Experiment 3: Manipulation of sequence predictability

Experiment 3 tested the influence of sequence predictability on temporal binding. We manipulated the order in which the spoken letters were presented on three levels: predictable, shuffled, and random. Sequence predictability is of interest, as on the one hand, better predictability might lead to increased use of strategies, e.g., always pressing the key at the same letter. On the other hand, reduced predictability might increase task load and derail attention from the visual task to the auditory timer. Finally, the movement of the visual Libet Clock is typically perfectly predictable (in fact we are not aware of a study that used randomly jumping pointer positions of a visual Libet clock). Finding that predictability of time markers did impact temporal binding might thus be an observation of general interest beyond the auditory timer employed here. Therefore, we tested how sequence predictability influences temporal binding.

### Methods

#### Participants

Forty-eight new participants (19 male, 8 left-handed) with a mean age of 26.10 years (*SD* = 7.30) who fulfilled the same criteria as in Experiments 1 and 2 took part in the experiment.

#### Apparatus and stimuli

The visual task was left unchanged from the first two experiments. For the auditory timer, participants again heard the German letters A, F, I, O, and T over headphones. In Experiment 3, we varied the predictability of the sequence in which the letters were presented on three levels (predictable, shuffled, and random) between blocks. The letter sequence in this experiment followed Experiments 1 and 2 in that intervals were 500 ms long and half-filled, which means they consisted of spoken letters with a length of 250 ms followed by 250 ms silence. In the predictable condition, participants repeatedly heard the letters A, F, I, O, and T, in the same order. In the shuffled condition, however, the order of the letter sequence was shuffled at the beginning of every trial. That is, participants could predict the letter sequence, but only on a trial basis and not for the whole experiment. In the random condition, the order of the letter sequence was also determined at the beginning of each trial, only this time the sequence was drawn randomly from the set of five letters, with the prerequisite that no letter could appear twice in a row.

#### Procedure

As the variable of interest in Experiment 3 was the sequence predictability, this factor was manipulated within subjects, and we divided the experiment into thirds and assigned a specific sequence predictability (predictable, shuffled, or random) to each third. The order of predictability types was counterbalanced across participants. The procedure for Experiment 3 followed that for Experiment 1, with two exceptions concerning the presentation of the scale for time estimations at the end of each trial.

In the predictable condition, the scale was the same as in the previous experiments; it started with the letter A and subsequently displayed the letters F, I, O, and T before finishing with another A so that all intervals between letters were displayed. As a new letter sequence was determined at the beginning of each trial in the shuffled condition, the scale had to be adjusted accordingly. In blocks with shuffled letter sequence, participants used a scale that displayed the respective letter sequence again with the starting and finishing letter being the same. The display of the scale in conditions with a random letter sequence was again different. In these trials, the scale was determined by displaying the actual timing (objectively correct judgment of the respective event) between the second and the fifth category. The surrounding letters were determined according to the sequence of the respective trial.

#### Design

The study used a 3 × 4 repeated-measures design with sequence predictability (predictable vs. shuffled vs. random) and condition (action experimental vs. action baseline vs. effect experimental vs. effect baseline) as within-subjects factors.

### Results

Data analysis in Experiment 3 followed that described in Experiment 1.

#### Temporal binding

Erroneous trials (0.6%) and outliers exceeding 2.5 SDs of the participant’s cell mean (3.3%) were excluded from the analyses.

##### Action binding

**Fig. 8:**
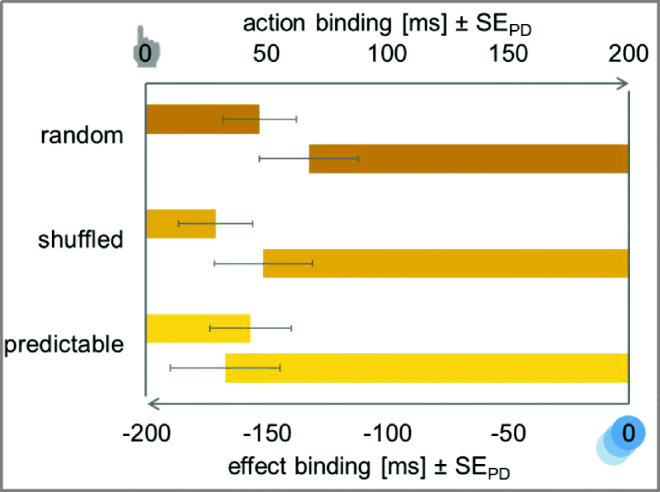
Temporal binding in Experiment 3. Action binding and effect binding relative to the baseline condition. The *y*-axis intercept denotes the perceived timing of the action (top) and the perceived timing of the effect (bottom) in the respective baseline conditions. Action binding is shown as bars from left to right to indicate the perceived delay of the action. Effect binding is shown as bars from right to left to indicate the perceived advancement of the effect. Error bars depict standard errors of paired differences for the factor sequence predictability (Pfister & Janczyk, 2013)

Separate *t*-tests revealed action binding in both the predictable condition, *t*(47) = 2.57, *p* = .013, *d*_*z*_ = 0.37, ∆ = 43.49 ms, and the random condition, *t*(47) = 3.10, *p* < .001, *d*_*z*_ = 0.45, ∆ = 47.29 ms. There was no clear evidence for or against action binding in the shuffled condition, *t*(47) = 1.90, *p* = .064, *d*_*z*_ = 0.27, *BF*_*10*_ = 1.36, ∆ = 29.08 ms. Participants judged actions in the predictable and random condition to be shifted towards the ensuing cursor movement (see Fig. 8).

The ANOVA for action binding with sequence predictability (predictable vs. shuffled vs. random) as within-subjects factor did not show any significant difference in the magnitude of action binding, *F* < 1, *BF*_*01*_ = 9.66.

##### Effect binding

Cursor movements in all three conditions were perceived to be shifted towards the preceding action, *t*_*p redictable*_(47) = −7.34, *p* < .001, *d*_*z*_ = 1.06, ∆ = −167.49 ms, *t*_*shuffled*_(47) = −7.43, *p* < .001, *d*_*z*_ = 1.07, ∆ = −151.76 ms, *t*_*random*_(47) = −6.43, *p* < .001, *d*_*z*_ = 0.93, ∆ = −132.88 ms. That is, cursor movements were perceived to have happened earlier when a keypress preceded this cursor movement.

The ANOVA for effect binding with sequence predictability (predictable vs. shuffled vs. random) as within-subjects factor did not show any significant differences between the different types of predictability, *F*(2,94) = 1.70, *p* = .188, η_p_^2^ = .04, *BF*_*01*_ = 3.54.

#### Explicit agency judgments

As in the first two experiments, there was only anecdotal evidence for a difference in judgments of agency between action experimental and effect experimental conditions, *F* < 1, *BF*_*10*_ = 2.44. Thus, explicit agency judgments were calculated across conditions. Again, agency ratings were high for all three types of judgment, authorship (*M* = 23.15, *SD* = 20.47), control (*M* = 22.20, *SD* = 20.74), and causation (*M* = 32.16, *SD* = 16.98).

**Fig. 9:**
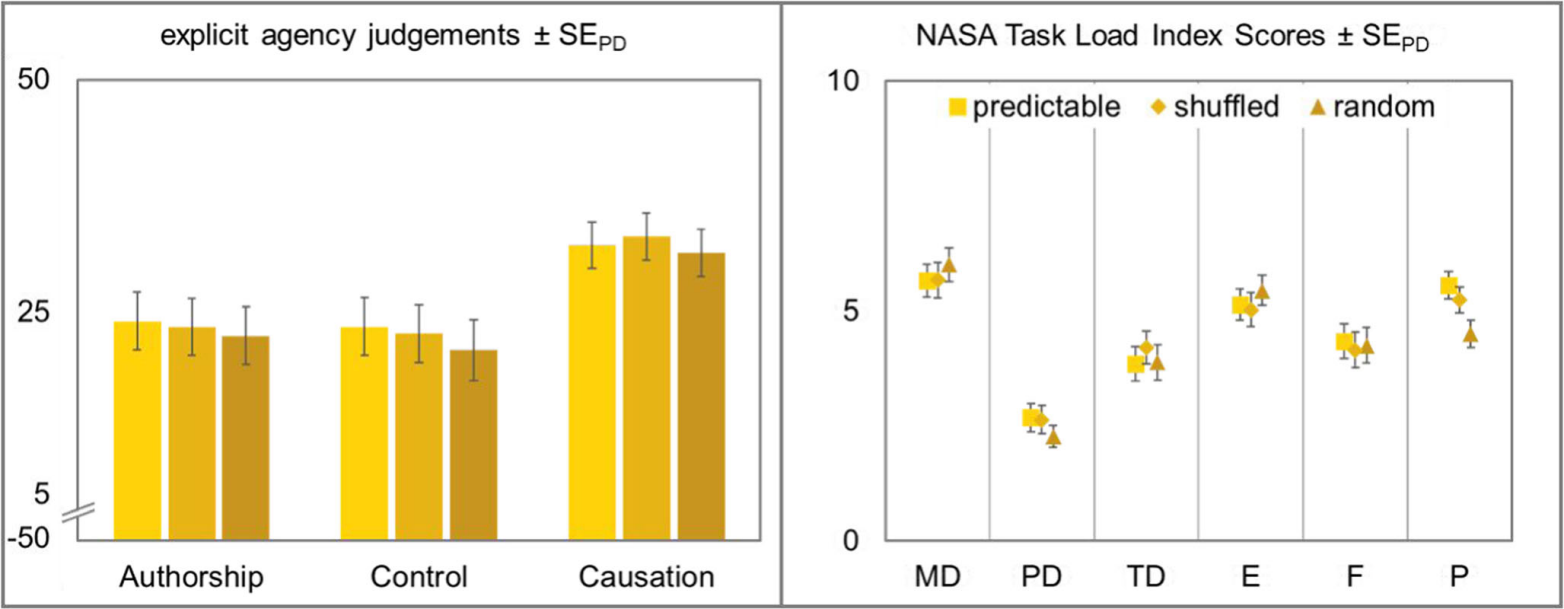
Left: Explicit agency judgments for authorship, control, and causation of the cursor movement. Agency judgments were made on a scale from −50 to 50 after every eighth trial in all experimental conditions. Right: Perceived task load as measured with the NASA Task Load Index (Hart & Staveland, 1988). MD: mental demand, PD: physical demand, TD: temporal demand, E: effort, F: frustration, P: performance. Squares represent participants’ judgments for perfectly predictable sequences, diamonds shuffled sequences which were determined at the beginning of each trial, and triangles for completely random sequences. All error bars depict standard errors of paired differences for the factor sequence predictability (Pfister & Janczyk, 2013)

Subsequently, three repeated-measures ANOVAs with sequence predictability (predictable vs. shuffled vs. random) as within-subjects factor were conducted. Explicit agency judgments did not differ significantly between the different sequence predictabilities, all *F*s < 1, all *BF*_*01*_ > 7.16 (see Fig. 9).

#### NASA Task Load Index

Data showed a significant effect of sequence predictability on performance, *F*(2,94) = 7.51, *p* = .001, η_p_^2^ = .14. There was no evidence for or against a difference between fully predictable and shuffled sequences, *t*(47) = 1.10, *p* = .278, *d*_*z*_ = 0.16, *BF*_*01*_ = 1.74. The random condition, however, elicited significantly lower performance ratings than both the predictable condition, *t*(47) = 3.91, *p* < .001, *d*_*z*_ = 0.56, and the shuffled condition, *t*(47) = 2.66, *p* = .011, *d*_*z*_ = 0.38. No other effects of sequence predictability were observed, *F*_*MD*_(2,94) = 1.09, *p* = .340, *BF*_*01*_ = 6.03, η_p_^2^ = .02, *F*_*PD*_(2,94) = 1.41, *p* = .248, η_p_^2^ = .03, *BF*_*01*_ = 4.54, *F*_*TD*_(2,94) = 1.28, *p* = .283, η_p_^2^ = .03, *BF*_*01*_ = 5.09, *F*_*E*_(2,94) = 1.03, *p* = .360, η_p_^2^ = .02, *BF*_*01*_ = 6.15, *F*_*F*_ < 1, *BF*_*01*_ = 11.66.

### Discussion

Experiment 3 served to examine whether the order in which the auditory stimuli are presented influences temporal binding. Therefore, we designed an experiment with three types of predictability of the letter sequences – predictable, shuffled, and random.

Similar to Experiment 1, we found temporal binding for both actions and events. However, there was no action binding in conditions with shuffled letter sequences. A comparison between the three types of sequence predictability nevertheless revealed no significant differences in temporal binding. Therefore, both the predictable sequence and the random sequence appear to be suitable for measuring temporal binding with our setup.

It is, however, worth noting that the presentation of the scales, which participants used to make their time judgments, differed between the conditions. This is a result of the study design, as participants always made their temporal judgments on a scale of 5+1 letters. While the scale in the predictable condition was always the same (AFIOTA), it changed in the other two conditions. For shuffled letter sequences, participants also saw a scale that had the same letter at the beginning and the end but was shuffled in between according to the sequence. Hence, participants had to adjust not only to a new letter sequence every trial but also to a newly arranged scale. Similar flexibility was demanded in the random condition, only this time participants saw only a snippet of the entire letter sequence which contained the objectively “correct” letters as well as at least one more element to the left and the right. Thus, scale presentation might have influenced participants’ performance and judgments in these conditions.

Surprisingly, sequence predictability had no notable influence on participants’ task load. They rated their task load to be about equally high in all three conditions. The only item that was influenced by sequence predictability was participants’ perceived performance. Participants rated their task completion as better in the predictable and shuffled condition compared to the random condition.

To sum up, implicit temporal binding measures suggest that either predictable or random letter sequences are suitable measures for temporal binding. Considering participants’ subjective ratings on performance, which tend to be lower for random sequences, gives an indication to using predictable or shuffled letter sequences. Therefore, we decided to stick with a predictable sequence for future studies.

## Experiment 4: Manipulation of sequence length

In Experiment 4, we systematically varied the sequence length, that is, how many different letters constitute the auditory timer. There were three different sequence lengths: 5 items, 10 items, and 15 items (for more detail see Apparatus and stimuli). As longer sequences should result in weaker retention of the sequence in working memory (cf. Miller, 1956), we expected both action binding and effect binding to decrease with increasing length of the letter sequence.

### Methods

#### Participants

Forty-eight new participants (12 male, 2 left-handed) with a mean age of 24.77 years (*SD* = 5.42) who fulfilled the same criteria as in the other three experiments were recruited.

#### Apparatus and stimuli

The visual task was left unchanged from the other experiments. For the auditory timer, participants again heard the German letters A, F, I, O, and T over headphones. Now, we varied the length of the letter sequence, that is, the number of letters in the sequence presented on three levels (5, 10, and 15) between blocks. The choice of these three levels was determined as follows: The smallest number of items should be easily remembered, as healthy humans can store at least 7 ± 2 items in their working memory (Miller, 1956). However, hearing the same five letters repeatedly might lead to frustration and boredom in the participants. Thus, we decided to present 10 letters as an intermediate level. These 10 letters were A, C, F, I, L, O, R, T, X, and Z. After a few trials, participants should be able to remember the presented letters without too much effort. In contrast, 15 letters should appear to be a random sequence to participants, as they will probably never hear the entire sequence during the trials. The 15-letter sequence consisted of the following letters: A, B, C, F, H, I, L, N, O, R, S, T, U, X, and Z.

**Fig. 10:**
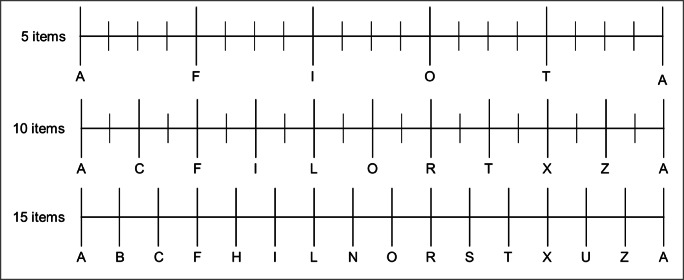
Scale presentation for temporal estimations in Experiment 4. The first row shows the scale presented when the auditory timer consisted of 5 items (1 px = 2.5 ms). In the middle the 10-items scale is presented (1 px = 5 ms), and at the bottom, the scale consisting of 15 letters (1 px = 7.5 ms)

As alluded to in the discussion of Experiment 3, an altered letter sequence carries the effect of a changing scale for temporal estimations as well. In the previous experiment, we addressed this issue by always presenting the same scale resolution while changing the anchors, i.e., letters on the scale. This time we decided to display the entire letter sequence at the end of every trial. Hence, in addition to the different levels of difficulty participants should have in remembering or getting attuned to the sequence, the resolution of the scale for temporal estimations decreased with increasing sequence length. The sequence lengths were set to 5, 10, and 15, so the scale for participants’ estimations would visually remain the same as more letters were added for the longer sequence lengths. While the visual appearance of the five-item scale was a scale with six anchors (AFIOTA) and three subdivisions each, the scale for 10 items displayed the respective 10 items plus the starting letter at the end. Each of these categories had one subdivision. The 15-item scale had no subdivisions and only displayed the 15+1 letters in sequence (see Fig. 10). These adjustments of the scale resulted in the following resolutions: one pixel on the 5-item scale equaled 2.5 ms, while one pixel on the 10-item scale was equal to 5 ms, and one pixel on the 15-item scale equal to 7.5 ms. Bottom line, during this experiment, participants always saw the entire sequence of letters when they gave their temporal estimation (see Fig. 10).

#### Procedure

As the variable of interest in Experiment 4 was the length of the letter sequence, this factor was manipulated within subjects, and we divided the experiment into thirds and assigned a specific sequence length (5, 10, and 15 letters) to each third. The order of sequence lengths was counterbalanced across participants. However, the manipulation of sequence length in this experiment involved changing the scale for time estimations as well. Apart from that, the procedure for Experiment 4 followed that described in Experiment 1.

#### Design

The study used a 3 × 4 repeated-measures design with sequence length (5 items vs. 10 items vs. 15 items) and condition (action experimental vs. action baseline vs. effect experimental vs. effect baseline) as within-subjects factors.

### Results

Data analysis in Experiment 4 followed that described in Experiment 1.

#### Temporal binding

Erroneous trials (0.4%) and outliers, trials in which temporal binding exceeded 2.5 SDs of the participant’s cell mean (3.1%), were excluded from the analyses.

##### Action binding

**Fig. 11:**
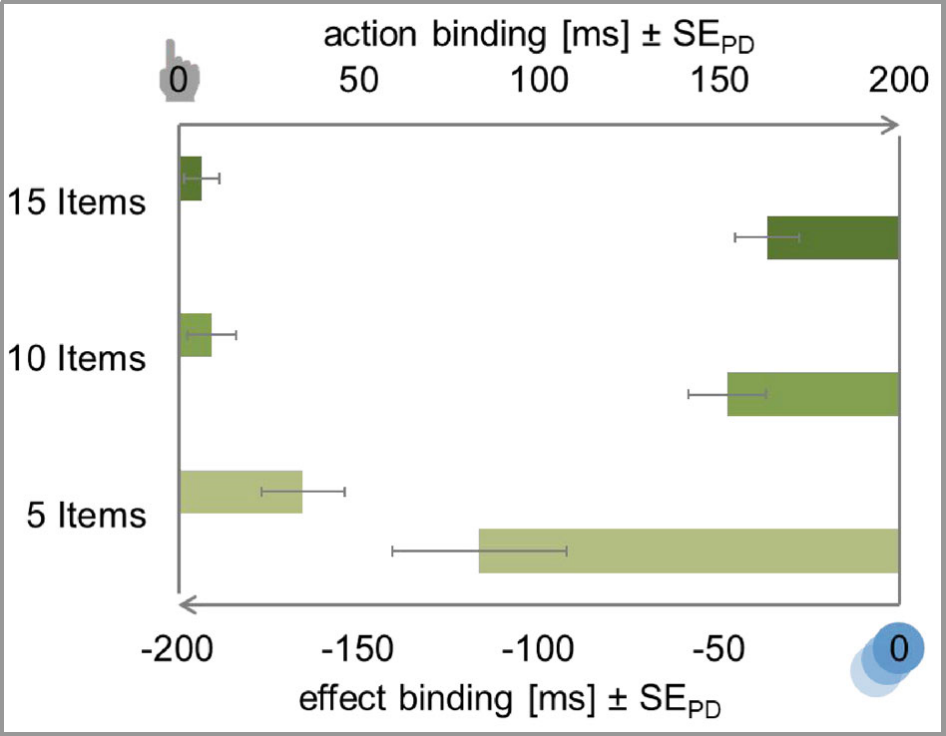
Temporal binding in Experiment 4. Action binding and effect binding relative to the baseline condition. The *y*-axis intercept denotes the perceived timing of the action (top) and the perceived timing of the effect (bottom) in the respective baseline conditions. Action binding is shown as bars from left to right to indicate the perceived delay of the action. Effect binding is shown as bars from right to left to indicate the perceived advancement of the effect. Error bars depict standard errors of paired differences for the factor sequence length (Pfister & Janczyk, 2013)

Participants judged their action to be shifted towards the effect only in blocks where the sequence consisted of five items (see Fig. 11). That is, actions were perceived to have happened later in the 5-item condition, *t*(47) = 3.02, *p* = .004, *d*_*z*_ = 0.44, ∆ = 34.56 ms, but not when the letter sequence consisted of 10 items, *t*(47) = 1.34, *p* = .186, *d*_*z*_ = 0.19, *BF*_*10*_ = 0.71, ∆ = 9.21 ms, or 15 items, *t*(47) = 1.33, *p* = .191, *d*_*z*_ = 0.19, *BF*_*10*_ = 0.70, ∆ = 6.55 ms (see Fig. 11).

The ANOVA for action binding with sequence length (5 items vs. 10 items vs. 15 items) as within-subjects factor revealed a significant difference in the magnitude of action binding between the three sequence lengths, *F*(2,94) = 3.75, *p* = .027, η_p_^2^ = .07. That is, action binding was significantly larger in the 5-item condition than in the 10-item condition, *t*(47) = 2.05, *p* = .046, *d*_*z*_ = 0.30. However, there was no clear evidence for or against a difference between the 10- and the 15-item conditions, *t* < 1, *BF*_*01*_ = 2.59.

##### Effect binding

Cursor movements in all three conditions were perceived to be shifted towards the preceding action, *t*_*5items*_(47) = −4.84, *p* < .001, *d*_*z*_ = 0.70, ∆ = −116.47 ms, *t*_*10items*_(47) = −4.48, *p* < .001, *d*_*z*_ = 0.65, ∆ = −47.75 ms, *t*_*15items*_(47) = −4.15, *p* < .001, *d*_*z*_ = 0.60, ∆ = −36.74 ms. That is, cursor movements were perceived to have happened earlier when a keypress preceded this cursor movement.

The ANOVA for effect binding with sequence length (5 items vs. 10 items vs. 15 items) as within-subjects factor showed a significant difference between the sequence lengths, *F*(2,94) = 13.32, *p* < .001, η_p_^2^ = .22. The temporal shift in perception was significantly larger in the 5-item condition than in the 10-item condition, *t*(47) = −3.61, *p* < .001, *d*_*z*_ = 0.52, while there was no clear evidence for or against a difference of effect binding in the 10- and 15-item conditions, *t*(47) = −1.18, *p* = .244, *d*_*z*_ = 0.17, *BF*_*01*_ = 1.63.

#### Explicit agency judgments

As in the other experiments, there was no significant difference in judgments of agency between action experimental and effect experimental conditions, *F* < 1, *BF*_*01*_ = 12.45. Thus, explicit agency judgments were calculated across conditions. Again, agency ratings were high for all three types of judgment, authorship (*M* = 22.20, *SD* = 23.78), control (*M* = 21.13, *SD* = 23.07), and causation (*M* = 35.37, *SD* = 13.96).

**Fig. 12:**
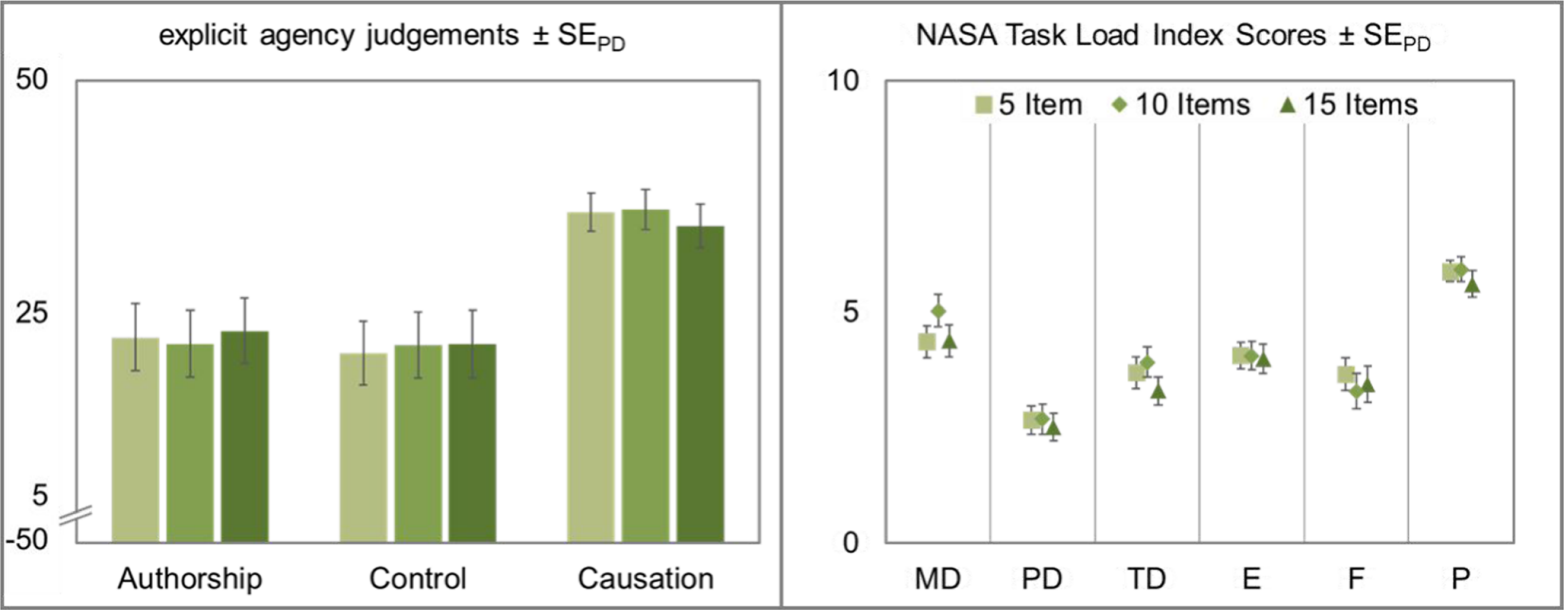
Left: Explicit agency judgments for authorship, control, and causation of the cursor movement. Agency judgments were made on a scale from −50 to 50 after every eighth trial in all experimental conditions. Right: Perceived task load as measured with the NASA Task Load Index (Hart & Staveland, 1988). MD: mental demand, PD: physical demand, TD: temporal demand, E: effort, F: frustration, P: performance. Squares represent participants’ judgments for sequences of 5 letters, diamonds 10 letters, and triangles for sequences of 15 letters. All error bars depict standard errors of paired differences for the factor sequence length (Pfister & Janczyk, 2013)

Subsequently, three repeated-measures ANOVAs with sequence length (5 items vs. 10 items vs. 15 items) as within-subjects factor were conducted. Explicit agency judgments did not differ significantly between the different sequence lengths, all *F*s < 1, all *BF*_*01*_ > 7.96 (see Fig. 12).

#### NASA Task Load Index

Data showed a significant effect of sequence length on temporal demand, *F*(2,94) = 3.26, *p* = .043, η_p_^2^ = .07. Temporal demand gradually increased with sequence length. However, it differed significantly only between the 5-item condition and the 15-item condition, *t*(47) = −2.66, *p* = .011, *d*_*z*_ = 0.38. Data did not provide clear evidence for or against a difference in mental demand between the three sequence lengths, *F*_*MD*_(2,94) = 2.91, *p* = .060, η_p_^2^ = .06, *BF*_*01*_ = 1.34. No other effects of sequence length were observed, all other *Fs* < 1, *BF*_*01*_ > 7.28.

### Discussion

In Experiment 4, we tested whether the sequence length of the auditory stimuli influences temporal binding. Therefore, we designed an experiment with three lengths of the letter sequences, 5 items, 10 items, and 15 items. Sequence length had a notable effect on both action binding and effect binding. Contrary to our hypothesis, participants showed action binding only in the 5-item condition, while effect binding, even though present in all three conditions, was drastically reduced for medium and long sequences. The implemented variation in sequence length carried the effect of an altered scale and scale resolution as well. Therefore, reduced temporal binding in the two longer sequence conditions could have resulted from the different scale presentation or participants’ estimation strategies. The absolute length of all three scales was equal; however, the 5- and 10-item scales had additional visual markers as subdivisions on the scale (see Fig. 10), making it possible to give more fine-grained estimations. While it was easy to predict the entire sequence in the 5-item condition, it was much harder for sequences consisting of 10 items, and almost impossible for the longest sequence of 15 items. We therefore suggest that participants might have tried to locate both the actions and effects with respect to both the preceding and ensuing letter in the 5-item condition. On the contrary, in the two longer sequence conditions, participants probably used only the previously heard letter as anchor for their estimation. Additionally, we presume that it is much easier to refer to experienced events than anticipated events, and therefore it is not surprising that estimations in the 10- and 15-item conditions show less variability than in the 5-item condition. Taken together, this accentuates the importance of using a scale which allows participants to give more fine-grained estimations.

## General discussion

With the present line of experiments, we investigated different design factors to establish an auditory measure for temporal binding. Specifically, we systematically manipulated four factors of the timed auditory letter sequence that served as auditory timer. These were interval length (250 ms, 500 ms, 750 ms), interval filling (filled, half-filled, sequenced), sequence predictability (predictable, shuffled, random), and sequence length (5 items, 10 items, 15 items). Overall, the setup that we used produced robust temporal binding for both actions and effects, which is crucial for the development of an alternative measure. Based on previous studies using a visual Libet Clock to measure temporal binding, both the absolute temporal binding and the standardized effect sizes we discovered were to be expected (e.g., Moore & Obhi, 2012; Ruess, Thomaschke, Haering, Wenke, & Kiesel, 2017a; Schwarz, Weller, Pfister, & Kunde, 2019b). If anything, effect binding seemed to be slightly larger than in previous studies, but it was consistent across all four experiments (*N* = 192) reported here. These observations make the auditory timer a potent means for measuring temporal binding, as it is possible to record participants’ perception of events timed to the millisecond. Recently, a new way of interpreting temporal binding in terms of multisensory cue integration has emerged (Kawabe, Roseboom, & Nishida, 2013; Legaspi & Toyoizumi, 2019; Lush et al., 2019). According to the authors, temporal binding can be explained by integrating and weighting information about planned actions and perceived sensory events. To make inferences about participants’ judgments, the method used for measuring temporal binding has to be precise, with high resolution. In line with this, we found that temporal binding was mostly influenced by the characteristics of the interval and the sequence length, and not so much by the presentation order of the letters. Consequently, the characteristics of the auditory timer should be adapted according to the research purpose. Single letters should be easy to discriminate, and the letter sequence should be of a length that can be displayed with a good spatial resolution on the screen, i.e., 1 px should account for only a few milliseconds of the auditory sequence.

Our attempt to use a previously employed auditory timer with an interval length of 250 ms (Cornelio Martinez et al., 2018) revealed higher task load and frustration compared to an interval length of 500 ms, which appeared to be a good interval length for letter discrimination. Additionally, this constitutes a cycle length of 2500 ms, which makes the auditory timer more comparable to the visual timer used in standard Libet Clock experiments (e.g., Schwarz, Burger, Dignath, Kunde, & Pfister, 2018; Schwarz, Weller, Klaffehn et al., 2019a; Weller, Schwarz, Kunde, & Pfister, 2017).

Another factor to be considered is whether the task configuration, i.e., a set goal, and the lack of freedom to choose an action influenced participants’ temporal estimations. Previous research concentrating on the influence of goal attainment on explicit judgments of agency found that goal attainment increased judgments of agency even if participants did not actually achieve the goal by themselves (Dewey, Seiffert, & Carr, 2010). In addition, Barlas, Hockley, and Obhi (2017) conducted a study in which participants either had to press a certain button or could freely choose from up to four different buttons. Results showed that freedom of choice increased both temporal binding and explicit agency judgments (see also Barlas & Obhi, 2013). In that light, our forced-choice setup may have reduced temporal binding, supporting the robustness of the present findings. Thus, the influence of the task setup is an interesting factor for future research.

Throughout all experiments, participants explicitly rated their sense of agency as high. Such high agency judgments might be due to the simplicity of the task; the cursor movement always followed participants’ keypresses, and the very low error rates show that participants had no difficulty in completing it. These observations are in line with previous research indicating that participants take credit even for successful events that they are not entirely responsible for (Dewey et al., 2010). These ratings did not differ between actions and effects. Schwarz, Weller, Klaffehn et al. (2019a) suggest that participants’ ratings for causation over outcomes should generally be higher than ratings over the responsibility for a distinct action. However, in their study, the questions that participants had to answer in blocks where the timing of the action had to be estimated were different from those in blocks in which the timing of the effect had to be estimated, whereas the questions in our experiments were the same in all blocks. Nevertheless, the importance of causality is supported by our observation that participants generally rate their causation higher than authorship and control. This is possibly due to the fact that from childhood on, healthy individuals make many assumptions about their causality on a daily basis, as the decision whether or not it was me comes fairly natural (Blakey et al., 2019; Wegner, 2003). On the contrary, we do not always reflect on our authorship and control over events when they happen as expected. Additionally, agency judgments in the present study reflect a general judgment of agency generated over eight trials, whereas implicit feelings of agency were recorded after each trial. The lack of variation in the explicit agency measures might also be explained by the idea that the implicit and explicit measures for sense of agency, i.e., temporal binding and agency judgments, probably rely on different mechanisms and therefore do not necessarily have to correlate (Dewey & Knoblich, 2014).

Note that all recommendations for the design of an auditory timer for measuring temporal binding are based on the particular task presented in this study, that is, moving a cursor through a 3 × 3 grid, and this was tested on an iPad only. Further research is necessary to investigate whether our conclusions generalize to other tasks and input devices. Until then, we suggest that the recommendations presented can be used to make informed design choices that affect the detection of any given effects to different extents. Therefore, every parameter should be selected carefully. Please do also note that the recommendations given above are not to be taken as the “gold standard” for designing any auditory timer; rather we grant that different methods are suitable for different research questions.

The paradigm that we used to elicit temporal binding is a rather basic task with a simple action and visual effect. Whether the setup is also suitable for even more visually demanding tasks needs to be further evaluated. Additionally, in the current study we only varied one factor at a time (except for Experiment 4, in which the manipulation of the sequence length was confounded with the resolution of the estimation scale), neglecting any possible interactions that might accompany certain design choices. We have briefly alluded to some of these possible interactions in the discussions of the respective experiments, e.g., how the length of the letter sequence influences scale presentation. Therefore, our design recommendations are specific for each design factor. Combinations of other manipulations might come with additional benefits or pitfalls.

To conclude, we found that most of the tested design choices were in principle able to detect temporal binding. Thus, the proposed auditory timer appears to be quite robust to variations within certain ranges and can be widely employed to study temporal binding for visually demanding tasks.

## Footnotes

1 Another question when employing such rather novel hardware for experimental setups pertains to their input lag, i.e., the systematic delay between the physical input and the device registering said input. Every technical device has input lag, and USB keyboards, which are typically used in experimental setups, come with an input lag of up to 8 ms. The devices that we used employed a touch sampling rate of 60 Hz, which equals up to 16 ms of input lag (even though newer devices improve on this). That said, in previous experiments we have successfully employed touchscreen devices in response time experiments (e.g., Dignath et al., 2020; Kunde, Schmidts, Wirth, and Herbort, 2017; Wirth, Dignath, Pfister, Kunde, and Eder, 2016a; Wirth, Kunde, and Pfister, 2019; Wirth, Pfister, Foerster, Huestegge, and Kunde, 2016b), showing high measurement precision with sufficient trials. Finally, as binding is computed as the difference between experimental and baseline conditions, which are both recorded using the same device, any systematic latencies should be cancelled out by subtraction.

2 Throughout this work, interval length describes the lengths of the letters used as auditory measure rather than the delay between two events in the experimental task. Action–outcome delay denotes the time frame between an action and a subsequent outcome.
